# Dynamics of pure tone audiometry and DPOAE changes induced by glycerol in Meniere’s disease

**DOI:** 10.1007/s00405-012-2246-6

**Published:** 2012-11-16

**Authors:** Agnieszka Jablonka-Strom, Lucyna Pospiech, Maciej Zatonski, Marek Bochnia

**Affiliations:** Otolaryngology Department, Faculty of Dentistry, Medical University of Wroclaw, ul. Borowska 213, 50-556 Wroclaw, Poland

**Keywords:** Glycerol test, DPOAE, Meniere’s disease, Endolymphatic hydrops, Otoacoustic emissions

## Abstract

The purpose of this study is to follow up the dynamics of pure tone threshold and DPOAE amplitude changes induced by glycerol with reference to its activity in the inner ear. Selection was made among 38 patients with Meniere’s disease for those having positive glycerol test. Pure-tone audiometry and DP-gram were performed in four series: as an initial examination before glycerol intake, 1, 2 and 3 h after. Audiometric changes formed distinct biphasal pattern at all frequencies between 250 and 4,000 Hz. The most dynamic pure tone threshold decrease occurred during the first hour. Between the first and second hour after glycerol ingestion there was a phase of no significant hearing changes. Further pure tone threshold decrease went on within the third hour reaching its top. Observing DPOAE changes, the highest DP amplitude growth occurred after the second and the third hour at DP-gram frequencies 2, 3 and 4 kHz. The fastest DP-amplitude increase was registered as well during the first hour after glycerol ingestion. In 11 persons with both audiometry and DPOAE positive glycerol test, parallel dynamics in the course of the glycerol test was observed. Biphasal glycerol test dynamics suggests the possibility of two mechanisms of glycerol activity in the inner ear.

## Introduction

Glycerol test, first introduced by Klockhoff and Lindblom in 1966 [1], is still common noninvasive diagnostic procedure in Meniere’s disease. Glycerol as strong osmolar agent rises the osmotic pressure of the liquid in which it is dissolved and induces diffusion process. Increased plasma osmolarity in blood, cochlear capillaries, stria vascularis causes endolymph diffusion into blood circulation reducing pressure and capacity of hydropic membranous labyrinth. Apart from passive endolymph flow, it is supposed that glycerol activates ionic pump within stria vascularis. Animals with surgically induced endolymphatic hydrops react to glycerol by intra- and extracellular oedema in stria vascularis [2]. Furthermore, glycerol increases the secretion of glycoproteins in the endolymphatic sac even in healthy animal ears [3]. Glycoproteins, by their osmotic properties, induce endolymph flow into endolymphatic sac where it is absorbed. Therefore, glycerol would activate endolymph absorption in radial direction, towards stria vascularis, and in longitudinal direction from the apex of the cochlear duct into the endolymphatic sac.

Standard glycerol test consists of pure tone audiometry and optional speech audiometry. Improvement in cochlear function after glycerol digestion expressed by hearing threshold decrease and better speech discrimination is described as a positive result of the glycerol test. Positive glycerol test is assumed to be an evidence of the endolymphatic hydrops. There have been some doubts concerning the test's accuracy, mainly within its sensitivity and subjectivness of pure tone audiometry [4]. Therefore, some effort have been made recently to improve the test and make its results more reliable. Glycerol test procedure modifications included some trials of applying additional inner ear tests as otoacoustic emissions [5, 6], electrocochleography [7, 8], ENG [9] or posturography [10]. Glycerol test in our studies, beside pure tone audiometry, is enriched by distortion products and otoacoustic emissions DPOAE to obtain more objective and specific results.

The purpose of the work was to follow up dynamics of pure tone threshold and DPOAE amplitude changes induced by glycerol in patients with Meniere’s disease that would explain potential mechanisms of glycerol activity in the inner ear.

## Materials and methods

The study population consisted of 38 patients identified with Meniere’s disease based on AAO-HNS 1995 criteria. There were 16 males and 22 females with a mean age of 48.2 years. Glycerol test was also performed in a control group consisting of 20 subjects (10 males and 10 females; mean age 44.8 years). All the subjects were given orally 86 % glycerol in 1.5 ml/kg of body weight, dissolved 1:1 in physiological saline. Pure tone audiometry and DPOAE were performed in four series: as an initial examination before glycerol intake (hour 0), 1, 2 and 3 h after the glycerol intake. Pure tone audiometry determined air conduction hearing thresholds on both ears separately and contralateral masking was performed whenever necessary. DPOAE was tested by ILO 88/92 otodynamics analyzer in DP-gram function, 1/2 octave steps, from 700 to 6,000 Hz according to *f*_2_, defined by 2*f*_1_ – *f*_2_ formula. Primary tones frequencies were in ratio *f*_2_/*f*_1_ = 1.22 and their intensity equal *L*_1_ = *L*_2_ = 70 dB SPL. Audiometric glycerol test was considered as positive when the hearing threshold lowered at least 15 dB at minimum three frequencies at any time of investigation after glycerol intake. DPOAE glycerol test was considered to be positive if: (a) DP amplitude increased above 5 dB at minimum 2 DP-gram frequencies or (b) initially absent otoemission appeared at three or more frequencies.

Among 38 Meniere patients, 18 subjects with positive audiometric glycerol test and 15 with positive DPOAE glycerol test were further analyzed. In each consecutive hour at each frequency, mean hearing threshold increment, mean DP amplitude increment, mean hearing threshold and mean DP amplitude were established. The term “increment” was applied to an increase or decrease of the hearing threshold or DP-amplitude with reference to the initial score before the glycerol administration. Increment is a basic unit exhibiting a change in time, i.e. hearing or DPOAE dynamics. Parametric Student's *t* test and ANOVA variation analysis were used in the statistics.

## Results

In the group of Meniere patients, audiometry positive glycerol test was reached by 18 subjects (47 %) and DPOAE positive test by 15 subjects (39 %). In the control group with cochlear hearing loss, audiometric glycerol test was positive in one subject (5 %) and DPOAE glycerol test in none. Another presentation of positive–negative results is showed in the Table 1.

**Table 1:** Distribution of positive and negative glycerol tests

	Positive glycerol test				Negative glycerol test in audiometry and DP
	Pure-tone audiometry (*n* = 18)			Total	
		DPOAE (*n* = 15)			
	Exclusively audiometry	Audiometry + DP	Exclusively DP		
Meniere patients (*n* = 38)	7	11	4	22 (58 %)	16 (42 %)
Control group (*n* = 20)	1	0	0	1 (5 %)	19 (95 %)

Four subjects presented audiometry positive test in both ears, giving 22 ears for further analysis of pure tone audiometry changes. Similarly, two subjects reached DPOAE positive test binaurally, yielding 17 ears for further analysis of DPOAE dynamics in the test course.

### Dynamics of pure tone audiometry changes

**Fig. 1:**
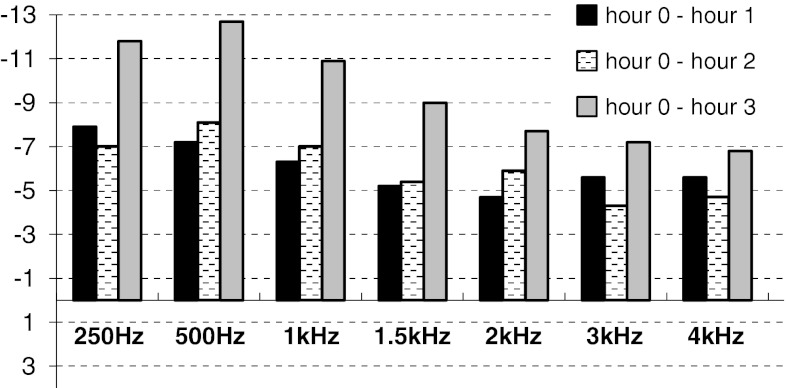
Mean hearing threshold increment in each consecutive hour in ears with positive glycerol test (dB). Baseline presents initial hearing threshold before glycerol administration

The most dynamic hearing improvement was observed within the first hour. Mean hearing threshold increment after the first hour ranged from −4.7 dB at 2 kHz to −7.9 dB at 250 Hz (Fig. 1). Second testing hour did not show any progression in hearing state, forming a kind of plateau phase. Third hour brought further hearing improvement. The most significant change in relation to initial value before glycerol administration was registered after the third testing hour at the frequencies below 1.5 kHz (mean increment ranging from −9.0 dB at 1.5 kHz to −12.7 dB at 500 Hz; *p* < 0.001) (Fig. 1). All mean hearing threshold increments at each frequency from 250 to 4,000 Hz in each testing hour were statistically significant (*p* < 0.001).

**Fig. 2:**
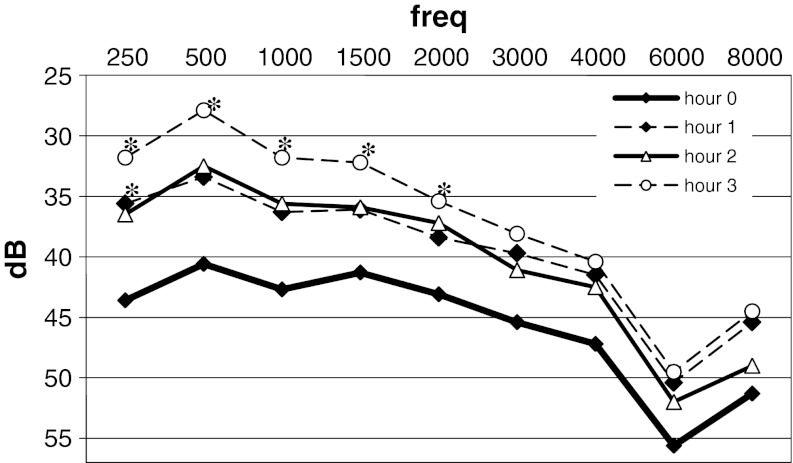
Mean hearing thresholds in each consecutive hour in ears with audiometry positive glycerol test (dB HL). *Asterisk* indicates a threshold significantly lower than the initial one (hour 0); *p* < 0.05

Mean hearing thresholds in the third testing hour at the frequencies from 250 to 2,000 Hz were significantly lower than mean hearing thresholds in the initial examination (Table 2; Fig. 2). Below 1 kHz, difference in their values was the most distinctive and amounted to approximately 12 dB. Hearing did not change by regular steady improvement but in two-step course. It is clearly visible in Figs. 1 and 2 that hearing was improving within the first and the third hour and was constant within the second hour.

**Table 2:** Mean hearing thresholds in each consecutive hour in ears with audiometry positive glycerol test (dB HL)

Hour	Frequency								
	250 Hz	500 Hz	1 kHz	1.5 kHz	2 kHz	3 kHz	4 kHz	6 kHz	8 kHz
Hour 0	43.6	40.6	42.7	41.3	43.1	45.4	47.2	55.6	51.3
Hour 1	***35.6***	33.4	36.3	36.1	38.4	39.7	41.5	50.4	45.4
Hour 2	36.5	32.5	35.6	35.9	37.2	41.1	42.5	52.0	49.0
Hour 3	***31.8***	***27.9***	***31.8***	***32.2***	***35.4***	38.1	40.4	49.5	44.5

Hearing threshold significantly lower than the threshold from initial examination (hour 0) was marked with the bold italic font

Positive result of the glycerol test was reached after the first hour since the glycerol intake in 8 ears and after the third hour in 15 ears. Pure tone average in hour 0 in the ears that reacted positively to glycerol was 43.1 dB HL and in those hydropic ears with negative test result was 53.0 dB HL.

### Dynamics of DPOAE alterations

**Fig. 3:**
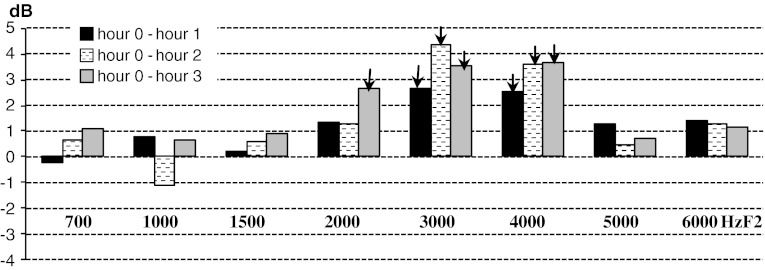
Mean DPOAE amplitude increments in each consecutive hour in ears with DPOAE positive glycerol test. Statistically significant increments are marked with an *arrow*

The largest DPOAE amplitude increments were registered after the second and third hour at 3 and 4 kHz (*f*_2_) (*p* < 0.01) (Fig. 3). The most dynamic DPOAE amplitude increase is observed within the first testing hours and at the DP-gram frequencies 2, 3 and 4 kHz.

**Fig. 4:**
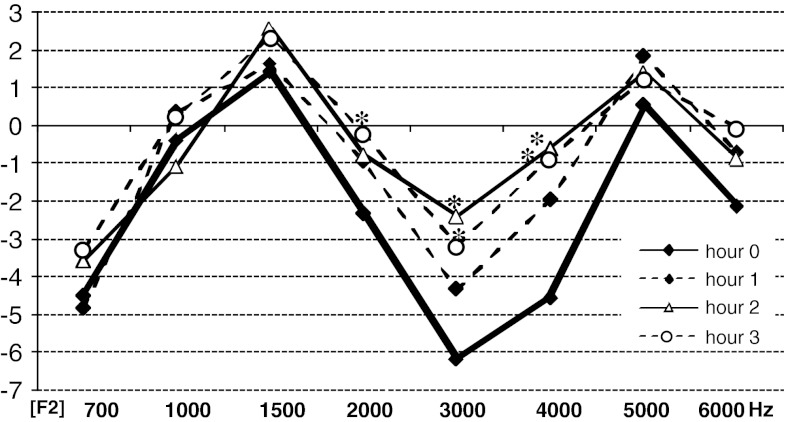
Mean DPOAE amplitude in each consecutive hour in ears with DPOAE positive glycerol test (dB SPL). *Asterisk* indicates an amplitude significantly higher than the initial one (hour 0)

Mean DPOAE amplitude at the frequencies 3 and 4 kHz (*f*_2_) after the second and the third hour is significantly higher than the one from the initial examination (hour 0 in Table 3 and Fig. 4) (*p* < 0.05). An analogical mean amplitude difference occurs at 2 kHz after the third hour. Mean DP-grams in each test hour are presented in Fig. 4.

**Table 3:** Mean DPOAE amplitude in each consecutive hour in ears with DPOAE positive glycerol test (dB SPL)

Hour	Frequency							
	700 Hz	1 kHz	1.5 kHz	2 kHz	3 kHz	4 kHz	5 kHz	6 kHz
Hour 0	−4.49	−0.42	1.41	−2.33	−6.17	−4.56	0.52	−2.13
Hour 1	−4.85	0.33	1.61	−0.96	−4.32	−1.99	1.83	−0.74
Hour 2	−3.60	−1.11	2.54	−0.80	***−2.42***	***−0.62***	1.38	−0.89
Hour 3	−3.30	0.23	2.32	***−0.23***	***−3.20***	***−0.88***	1.21	−0.09

DPOAE amplitude significantly higher than the amplitude from initial examination (hour 0) was marked with the bold italic font

DPOAE glycerol test was considered as positive after one hour since glycerol ingestion in 10 ears, after 3 h in 7 ears. In the initial examination, average DPOAE amplitude (calculated from the values at all DP-gram frequencies) in ears which further reached positive DPOAE glycerol test was −2.2 dB SPL and in affected ears which demonstrated negative test results was −5.9 dB SPL. Mean DPOAE amplitude increments in the positive test ears differed significantly from the increments in the ears from the control group at each frequency from 1 to 6 kHz (*f*_2_) (3.4 kHz, *p* < 0.001; 2 kHz, *p* < 0.01; 1, 1.5, 5, 6 kHz, *p* < 0.05).

In 11 subjects that presented both audiometry and DPOAE positive glycerol test, parallel dynamics in both test was observed. Five of them developed an audiometry and DPOAE positive test result in the first hour, the next six in the third hour.

## Discussion

The studies revealed a kind of biphasal schema in the glycerol test dynamics. Both in the pure tone audiometry and in DPOAE, the most dynamic improvement occurred in the first test hour. The most significant hearing threshold increments and the greatest difference in mean hearing thresholds in relation to the initial values are observed in the last testing series, i.e. after the third hour from glycerol intake. In DPOAE, the largest increments and the highest amplitude is registered after the second and, as in the audiometry, after the third test hour. Thus both pure tone audiometry and DPOAE showed the best results after the third test hour. Particularly, pure tone audiometry outcomes highlighted two-stage test course by presenting significant improvement within the first and the third test hour and transitional phase within the second hour with no changes at all. It is worth mentioning that 11 subjects with both positive test result, audiometry and DPOAE, performed similar dynamics of the test course in the former and the latter. This biphasal schema of the test dynamics would implicate two mechanisms of glycerol activity in the inner ear, e.g. active and passive.

There are few reports concerning dynamics of audiological parameter alterations in the course of the glycerol test. Karjalainen et al. [4] observed significant hearing and speech discrimination improvement two and half an hour after glycerol ingestion. Klockhoff set hearing threshold every hour in 7 consecutive hours since glycerol intake. His observations coincided with ours that maximum effect had been reached after the third hour and the most dynamic hearing improvement occurred within the first hour [1]. He suggested that this early phase of the glycerol test might result from osmotic inhibition of endolymph secretion or direct osmotic impact on the Corti organ [1].

In microscopic study, Filipo et al. observed cellular swelling in stria vascularis after glycerol intake [2]. Together with this finding, Rask-Andersen et al. [3] noted increased post-glycerol secretion of high osmolar hydrophilous glycoproteins in endolymphatic sac. Perhaps in our study, during the first hour, following the osmotic pressure increase in the blood, stria vascularis and perilymph, dehydratation proceeded radially, i.e. on the whole surface of the endolymphatic membrane. Further, in the third hour, dehydratation could proceed longitudinally towards endolymphatic duct and sac. Immunocytochemical studies on hydropic guinea pigs’ ears, performed by Meyer zum Gottesberge, showed two places of glycerol uptake: tectorial membrane and endolymphatic sac [11]. She suggested two active mechanisms of glycerol activity in the inner ear. One would be modulation of sound proceeding in the inner ear by structural reorganization of the tectorial membrane. The second mechanism would be an influence on glycoprotein secretion in endolymphatic sac and thereby endolymph absorption activation in its area [11].

Some assumption could be put forward as an explanation of biphasal nature of the glycerol test. Firstly, perhaps passive and active glycerol activities coexist that are shifted in time. The former consists in simple osmotic diffusion, the latter–in activation of ionic pump in the stria vascularis. Alternatively, it could be assumed that during the first hour glycerol induces radial endolymph absorption in stria vascularis and during the third hour longitudinal flow leads to increased absorption in the endolymphatic sac. This subsequent process of the longitudinal flow could be due to secondary glycerol presence in cerebrospinal fluid [12] or due to increased glycoprotein secretion in the endolymphatic sac induced by glycerol as observed by Rask-Andersen [3].

DPOAE pattern of changes is not as distinct as biphasal audiometry pattern. Actually, hearing threshold and DPOAE outcomes are not in very straight correlation. Glycerol-induced alteration in DPOAE as a result of two or more mechanisms might be shifted in phase. Phase detection method in DPOAE would be of value in better demonstrating the dynamics of DPOAE changes. Mom et al. [13] observed post-glycerol OAE phase shift at 1 kHz with no significant magnitude changes. He interpreted it by glycerol role in releasing stapes plate mobility, initially blocked by overpressure in the perilymph, with little impact on OHC functioning. Valk [14] who also registered DPOAE phase shift after induction of acute endolymphatic hydrops in guinea pig, claimed that it is the result of changes in basilar membrane stiffness.

Frequency range of significant hearing improvement extended from 250 to 4,000 Hz, but it was the most evident between 250 and 2,000 Hz. Hearing improvement at lower frequencies is well known to researchers and results from properties of the endolymphatic hydrops that grows from the cochlear apex, an area of low frequencies perception. Mori also observed the most significant hearing threshold decrease at frequencies below 2,000 Hz [7]. Glycerol test on a large Meniere’s patients group, performed by Snyder [15], also revealed the best hearing improvement within lower frequencies. In our studies, DPOAE changes induced by glycerol are located in middle frequencies from 2,000 to 4,000 Hz according to *f*_2_. Within this frequency range in DP-gram, initial measurement before glycerol intake had showed that the lowest DP amplitude rised most significantly in the course of the test. Just the frequency of 2,000 Hz showed the best correlation between audiometry and DPOAE alteration in each consecutive test hour. According to Magliulo and Cianfrone, the exact DP frequencies—2,000–4,000 Hz—showed significant increase of DP threshold in growth rate function after surgical induction of the endolymphatic hydrops in guinea pigs. This DP deterioration is also best reversed at these frequency ranges after glycerol intake by guinea pigs [8]. Okubo et al. pointed out DP amplitude decrease at 4,000 and 6,000 Hz in hydropic guinea pigs’ ears and following increase after isosorbide administration. Electrocochleography applied by Okubo seemed to be less sensitive tool in such monitoring [16]. Summating potentials (SP) are known to be enhanced in endolymphatic hydrops although possible SP decreasing induced by glycerol remains unclear. Some authors reported tendency to SP normalization at lower frequencies after glycerol dehydration [17]. Others observed similar influence (decrease in SP/AP ratio) exclusively after intravenous glycerol administration, as opposed to oral administration [18]. More recent investigations performed with extratymphanic electrocochleography revealed no glycerol impact either on elevated SP or SP/AP ratio in hydropic ears [19]. It might be concluded that the function of hair cells monitored by electrocochleography is not so directly linked to displacement of the basilar membrane produced by growing endolymphatic hydrops on one side or by glycerol dehydratation on the other side. TEOAE were also observed to be altered in the glycerol test. Kubo et al. [20] noted that endolymphatic hydrops decompression resulted in dominating TEOAE otoemission shift toward higher frequencies.

Changes at 6 and 8 kHz in the glycerol test did not correspond to the changes at other frequencies. High pure tone threshold fluctuations in the course of the test in individual subjects caused high standard deviations and no significant alteration. Some authors emphasized that the test should be assumed as positive at appropriate hearing improvement only within lower frequencies [15, 21]. It is difficult to find a proper explanation for the pathophysiology of these fluctuations. Reception locum for these frequencies lies in the basic cochlear turn, closer to the vestibule and endolymphatic duct. Maybe quicker endolymph flow evoking a phenomenon of endolymph inertia plays a role. Paparella suggested that Meniere patients had a different hearing loss mechanism at lower frequencies in comparission to higher frequencies. Perhaps hearing loss at the cochlear apex is a result of excessive shearing forces and at the base–hair cell exposition to potassium following endolymphatic sac ruptures [22].

There are still many doubts to be explained in glycerol impact to hydropic ears and pathophysiology of endolymphatic hydrops itself.

## Summary

Audiometry and DPOAE measurements in the glycerol test procedure are the most profitable after the third hour since glycerol administration due to the most significant outcomes of both at this time. The most dynamic improvement of audiometric and DP parameters occurs during the first test hour. Analysis of dynamics shows two-step course of the glycerol test with pure tone audiometry improvement within the first and third hour and no progress within the second hour. Observed changes in pure tone audiometry concern lower frequencies and in DPOAE, middle frequencies.

Biphasal glycerol test dynamics suggests a possibility of two glycerol activity mechanisms in the hydropic inner ear.

## References

[1] Klockhoff I, Lindblom U (1966). Glycerol test in Meniere’s disease. Acta Otolaryngol (Stockh).

[2] Filipo R, Barbara M, Mancini P, Coletti A, Attanasio G, Modesti A, Sterkers O, Ferrary E (2000). New insights into glycerol in experimental endolymphatic hydrops. Meniere’s disease 1999—update.

[3] Rask-Andersen H, Friberg U, Erwal C, Jansson B (1989). Effects of hyperosmolar substances on the endolymphatic sac. Acta Otolaryngol (Stockh).

[4] Karjalainen S, Karja J, Nuutinen J (1984). The limited value of the glycerol test in Meniere’s disease. J Laryngol Otol.

[5] Cianfrone G, Ralli G, Fabbricatore M, Altissimi G, Nola G (2000). Distortion product otoacoustic emissions in Meniere’s disease. Scand Audiol.

[6] Inoue Y, Kanzaki J, O-Uchi T, Ogawa K (1997). Clinical application of transiently evoked otoacoustic emissions after glycerol administration for diagnosis of sensorineural hearing loss. Auris Nasus Larynx.

[7] Mori N, Asai A, Suizu Y, Ohta K, Matsunaga T (1985). Comparison between electrocochleography and glycerol test in the diagnosis of Meniere’s disease. Scand Audiol.

[8] Magliulo G, Cianfrone G, Musacchio A, Vingolo GM, Petti R, Cristofari P (1997). Distortion-product otoacoustic emissions and glycerol on the guinea pig hydropic ear. J Otolaryngol.

[9] El-Gohary MAH, Kamal NI, El-Kahky AM, Taha HM, Sterkers O, Ferrary E (2000). Vestibulo-cochlear response to glycerol in Meniere’s patients. Meniere’s Disease 1999—update.

[10] Di Girolamo S, Picciotti P, Sergi B, D’Ecclesia A, Di Nardo W (2001). Postural control and glycerol test in Meniere’s disease. Acta Otolaryngol (Stockh).

[11] Meyer zum Gottesberge AM, Mai JK, Sterkers O, Ferrary E (2000). Dual effect of glycerol administration on glycoconjugates in the adult guinea pig inner ear. Meniere’s disease 1999—update.

[12] Gosepath K, Maurer J, Amedee R, Mann W (1997). Tympanic membrane displacement analysis after glycerol intake: effects on intracranial and intracochlear fluid pressure. Am J Otol.

[13] Mom T, Gilain L, Avan P (2009). Effects of glycerol intake and body tilt on otoacoustic emissions reflect labyrinthine pressure changes in Meniere’s disease. Hear Res.

[14] Valk WL, Wit HP, Albers FWJ (2004). Evaluation of cochlear function in an acute endolymphatic hydrops model in the guinea pig by measuring low-level DPOAEs. Hear Res.

[15] Snyder JM (1982). Predictability of the glycerin test in the diagnosis of Meniere’s disease. Clin Otolaryngol All Sci.

[16] Okubo H, Tachihara N, Satoh S, Hara A, Kusakari J (1997). Effect of isosorbide on distortion-product otoacoustic emissions and endocochlear DC potential in experimentally induced hydropic ears. Acta Otolaryngol (Stockh).

[17] Dauman R, Aran JM, Charlet de Sauvage R, Portmann M (1988). Clinical significance of the summating potential in Menière’s disease. Am J Otol.

[18] Aso S, Watanabe Y, Mizukoshi K (1991). A clinical study of electrocochleography in Menière’s disease. Acta Otolaryngol.

[19] Takeda T, Kakigi A (2010). The clinical value of extratympanic electrocochleography in the diagnosis of Ménière’s disease. ORL J Otorhinolaryngol Relat Spec.

[20] Kubo T, Sakashita T, Kusuki M, Nakai Y (1995). Frequency analysis of evoked otoacoustic emissions in Meniere‘s disease. Acta Otolaryngol (Stockh).

[21] Sauer RC, Kaemmerle AW, Arenberg JK (1980). The prognostic value of the glycerol test. Otolaryngol Clin N Am.

[22] Paparella MM (1991). Patogenesis and pathophysiology of Meniere’s disease. Acta Otolaryngol (Stockh).

